# Fecal Concentrations of Long-Chain Fatty Acids, Sterols, and Unconjugated Bile Acids in Cats with Chronic Enteropathy

**DOI:** 10.3390/ani13172753

**Published:** 2023-08-30

**Authors:** Chi-Hsuan Sung, Rachel Pilla, Sina Marsilio, Betty Chow, Kailee A. Zornow, Jennifer E. Slovak, Jonathan A. Lidbury, Joerg M. Steiner, Steve L. Hill, Jan S. Suchodolski

**Affiliations:** 1 Gastrointestinal Laboratory, Department of Small Animal Clinical Sciences, Texas A&M University, College Station, TX 77843, USA; csung@cvm.tamu.edu (C.-H.S.);; 2 UC Davis School of Veterinary Medicine, Department of Veterinary Medicine and Epidemiology, University of California, Davis, CA 95616, USA; 3 Veterinary Specialty Hospital, San Diego, CA 92121, USA; 4 VCA Animal Specialty and Emergency Center, Los Angeles, CA 90025, USA; 5 The Schwarzman Animal Medical Center, New York, NY 10065, USA; 6 Flagstaff Veterinary Internal Medicine Consulting, Flagstaff, AZ 86004, USA

**Keywords:** fecal metabolome, chronic inflammatory enteropathy, lipid metabolism, inflammatory bowel disease, low-grade intestinal T-cell lymphoma, small cell lymphoma

## Abstract

**Simple Summary:**

Chronic enteropathy (CE) is a spectrum of chronic digestive disorders in cats. Understanding the metabolic dysfunction in the gut is crucial for understanding these diseases and developing better treatment options. However, the specific metabolic profiles of cats with CE remain largely unexplored. As fat is an essential energy source that participates in many physiological pathways, we focused on fat-related metabolites in this study. Fecal samples from cats with CE and healthy cats were collected, and concentrations of various fatty acids, sterols, and bile acids were measured. Cats with CE had higher concentrations of fatty acids, increased concentrations of animal-based sterols, and decreased plant-based sterols in their feces. A subset of cats with CE also showed abnormal bile acid metabolism, i.e., an increased percentage of primary bile acids. These findings suggest that cats with CE may have altered fat metabolism in their digestive tracts.

**Abstract:**

Chronic enteropathy (CE) in cats encompasses food-responsive enteropathy, chronic inflammatory enteropathy (or inflammatory bowel disease), and low-grade intestinal T-cell lymphoma. While alterations in the gut metabolome have been extensively studied in humans and dogs with gastrointestinal disorders, little is known about the specific metabolic profile of cats with CE. As lipids take part in energy storage, inflammation, and cellular structure, investigating the lipid profile in cats with CE is crucial. This study aimed to measure fecal concentrations of various fatty acids, sterols, and bile acids. Fecal samples from 56 cats with CE and 77 healthy control cats were analyzed using gas chromatography-mass spectrometry, targeting 12 fatty acids, 10 sterols, and 5 unconjugated bile acids. Fecal concentrations of nine targeted fatty acids and animal-derived sterols were significantly increased in cats with CE. However, fecal concentrations of plant-derived sterols were significantly decreased in cats with CE. Additionally, an increased percentage of primary bile acids was observed in a subset of cats with CE. These findings suggest the presence of lipid maldigestion, malabsorption, and inflammation in the gastrointestinal tract of cats with CE. Understanding the lipid alterations in cats with CE can provide insights into the disease mechanisms and potential future therapeutic strategies.

## 1. Introduction

Feline chronic enteropathy (CE) is among the most common disorders in cats, characterized by gastrointestinal signs persisting for at least three weeks [1]. Chronic inflammatory enteropathy (CIE, also known as inflammatory bowel disease (IBD)) and low-grade intestinal T-cell lymphoma (LGTCL, previously named alimentary small cell lymphoma, SCL) are the most common forms of feline CE. Although the etiology of feline CE is poorly understood, feline CE is considered multifactorial, where genetic, environmental, and immunological factors, as well as the intestinal microbiome, all play a role. Studies have described shifts in the gut microbiota composition of cats with CE [2,3,4,5,6]. However, research on the fecal metabolome in cats with CE remains limited [7].

Fecal metabolic profiles can provide an overview of the health of the gastrointestinal tract [8,9]. Overall, fecal metabolites illustrate biological pathways involving both the host and the gut microbiota. The contributions of the host’s metabolism, dietary intake, and microbial activities collectively shape the profile of these metabolites. Studies have shown that dogs with CE have significant changes in their fecal and serum metabolome, such as bile acids (BAs), amino acids, short- and long-chain fatty acids (SCFA and LCFA), vitamins, and their derivatives [10]. Among these metabolites, fatty acids (FAs), sterols, and BAs are related to lipid metabolism. Lipid metabolism is essential for energy balance and is involved in chronic inflammation and signal transduction. 

LCFAs have various functions in the body, such as being components of cell membranes, serving as an energy source, or as signaling molecules. Receptors for LCFAs are widely distributed on intestinal cells, in the pancreas, and in adipose tissue [11,12,13]. Additionally, receptors for LCFAs can also be found on macrophages, T cells, and neutrophils, indicating their interaction with the immune system [12,14]. Cholesterol, the main sterol in animals, is an essential component of cell membranes and functions as a precursor of oxysterols; Bas; fat-soluble vitamins; and steroid hormones such as cortisol, estradiol, progestins, and testosterone. Primary BAs, including cholic acid (CA) and chenodeoxycholic acid (CDCA), are synthesized from cholesterol by hepatocytes [15,16]. In cats, primary BAs are conjugated almost exclusively with taurine [17] before being secreted into the duodenum, where they help emulsify dietary lipids, mainly comprised of LCFAs, into small lipid droplets that can be broken down into long- or medium-chain FAs by pancreatic lipase. Conjugated primary BAs are deconjugated by a wide range of bacterial species. A narrow range of bacteria, including *Peptacetobacter* (former *Clostridium*) *hiranonis*, converts primary BAs into secondary BAs, including deoxycholic acid (DCA), lithocholic acid (LCA), and ursodeoxycholic acid (UDCA) [18]. Secondary BAs have anti-inflammatory and antimicrobial effects [19,20,21], which help in modulating intestinal functions. 

Studies have reported altered fecal concentrations of FAs, sterols, and BAs in humans with gastrointestinal disease [22,23] and dogs with CE [24]. However, limited research has been conducted on lipid metabolism in cats with CE [7]. To better understand the biological roles of these molecules, targeted approaches using validated assays are needed. Such assays may provide insights into the pathophysiology of CE and could help evaluate the impact of dietary and other therapeutic interventions. Therefore, our study aimed to compare the concentrations of selected fecal metabolites between cats with CE and healthy cats using a targeted, validated assay. 

## 2. Materials and Methods

### 2.1. Sample Population

Archived fecal samples collected between 2016 and 2021 from 77 clinically healthy cats and 56 cats with CE were retrospectively analyzed [3,4,7,25]. These samples were from three different cohorts, in which cats were under veterinary care and treated for CE at the Small Animal Hospital at Texas A&M University (College Station, TX, USA), at the Veterinary Specialty Hospital of San Diego (San Diego, CA, USA), and at the Schwarzman Animal Medical Center (New York, NY, USA). All cats were client-owned and were fed various commercial pet foods or home-prepared diets. Cats with chronic gastrointestinal signs (i.e., vomiting, diarrhea, weight loss, and/or hyporexia/anorexia of at least three weeks in duration) and histopathologic evidence of mucosal inflammation or neoplastic cell infiltration were assigned to the CE group (total: *n* = 56; from Texas: *n* = 6, California: *n* = 18, and New York: *n* = 32). The final diagnosis of IBD (CIE) or LGTCL was based on the evaluation of a histopathological examination of biopsy specimens by board-certified veterinary pathologists. Immunohistochemistry staining (for CD3 expressed by T cells and/or CD79a, CD20, or PAX-5 expressed by B cells) and/or PCR for antigen receptor rearrangement was performed, where additional diagnostics were required to reach a diagnosis, as described previously [3,4,7]. Cats with CE that received antibiotics or acid suppressants within 4 weeks were excluded at Texas A&M University Small Animal Hospital and the Veterinary Specialty Hospital of San Diego. Similarly, cats with CE that received antibiotics or acid suppressants within 2 weeks prior to enrollment were excluded at the Schwarzman Animal Medical Center.

A total of 77 clinically healthy cats, comprising 53 from Texas, 8 from California, and 16 from New York, were included in the healthy group. The health status of the cats from Texas and California was verified through an owner questionnaire on general and gastrointestinal health [3]. Cats that had received antibiotics, antacids, anti-inflammatory drugs, or corticosteroids within the past 6 months were excluded at the Texas A&M University Small Animal Hospital and the Veterinary Specialty Hospital of San Diego. Details of the healthy control cats from the Schwarzman Animal Medical Center were previously described [25].

Owners were instructed to collect naturally passed feces in the home environment within 24 h of defecation. Fecal samples were fridged (4 °C) or frozen (−20 °C) after being collected by the owner [3,25]. The fecal samples were frozen (−80 °C) at the Schwarzman Animal Medical Center and the Veterinary Specialty Hospital of San Diego until a batch shipment with dry ice to the Gastrointestinal Laboratory at Texas A&M University. Samples were stored at −80 °C prior to analysis at the Gastrointestinal Laboratory at Texas A&M University. Written informed client consent was obtained before the enrollment of each cat, and the study protocol was approved by the Institutional Animal Care and Use Committee at Texas A&M University (IACUC 2015-0276 and IACUC 2021-0035), the Schwarzman Animal Medical Center (IACUC 11-25-20), and the Research Advisory Committee at the Veterinary Specialty Hospital of San Diego.

### 2.2. Quantification of Fecal Long-Chain Fatty Acids, Sterols, and Unconjugated Bile Acids

The targeted compounds included 12 LCFAs, 10 sterols, and 5 unconjugated BAs. The selection of these metabolites was based on two primary reasons. Firstly, these compounds were found altered in our previous study using an untargeted metabolome approach in a subset of the current population [7]. Secondly, the analytical proficiency of our laboratory, specifically gas chromatography coupled with mass spectrometry (GC-MS), allowed us to develop this assay modified from published studies [26,27].

#### 2.2.1. Chemicals and Reagents

All reagents and solvents were of analytical reagent grade or maximum available purity. The standards of FAs, sterols, and BAs were obtained from Millipore Sigma (St. Louis, MO, USA), ChromaDex (Irvine, CA, USA), and Steraloids (Newport, RI, USA). Deuterated internal standards (d_4_-cholestane, d_6_-cholesterol, d_7_-sitostanol, d_4_-stearic acid, d_4_-cholic acid, and d_4_-lithocholic acid) were obtained from C/D/N Isotopes Inc. (Pointe-Claire, QC, Canada). The derivatization agent, Sylon HTP (a mixture of hexamethyldisilazane, chlorotrimethylsilane, and pyridine = 2:1:10 (*v*/*v*/*v*)), was purchased from Millipore Sigma. Concentrated hydrochloric acid (HCl, 37%), 1-butanol, chloroform, and hexane of HPLC-grade purity were purchased from Sigma-Aldrich or VWR (Radnor, PA, USA). 

#### 2.2.2. Stock Solutions and Calibration Standards

The stock solutions of FAs (2 mg/mL) and sterols (2 mg/mL) were prepared in chloroform and stored at −20 °C. The stock solutions of BAs (1 mg/mL) were prepared in 1-butanol and stored at −20 °C. The calibration standards were prepared as a series of two-fold dilutions with physiologically relevant concentrations in chloroform or 1-butanol. The internal standards were prepared at 2 mg/mL in chloroform (d_4_-cholestane, d_6_-cholesterol, d_7_-sitostanol, and d_4_-stearic acid) or hexane (d_4_-cholestane) and at 1 mg/mL in 1-butanol (d_4_-cholic acid and d_4_-lithocholic acid) and stored at −20 °C.

#### 2.2.3. Sample Preparation

Approximately 1000 mg of a fecal sample was aliquoted and frozen overnight at −80 °C, then lyophilized overnight, and finally homogenized with a disposable flexible plastic spatula. An aliquot of 10–14 mg of each lyophilized fecal sample was weighed in a 7 mL glass centrifuge tube for further sample processing. The weight of each sample was recorded for the final concentration calculation. A mixture of 160 μL 1-butanol; 10 μL each of the internal standards, including d_7_-sitostanol, d_6_-cholesterol, d_4_-stearic acid, and d_4_-cholestane (2 mg/mL each); and 20 μL each of d_4_-cholic acid and d_4_-lithocholic acid (1 mg/mL each) was added to each sample. A volume of 20 μL of concentrated HCl was added to each tube, which was then vortexed for at least 30 s; the tube was then incubated at 65 °C for 4 h in a heating station (Thermo Scientific, MA, USA, REACTI-Therm III #TS-18824 Heating module). After incubation, the samples were briefly vortexed, then dried with heat under nitrogen gas until visibly dry. Two hundred microliters of a commercial silylating mixture (Sylon HTP) were added to each dried tube for 30-min incubation at 65 °C. After incubation, the tubes were briefly vortexed before drying under heat with a nitrogen flow. Two hundred microliters of hexane were added to each tube, and the tubes were briefly vortexed. The tubes were then centrifuged at 18.0× *g* force at 5 °C for 10 min (Eppendorf Centrifuge 5810 R). A 50 μL sample of supernatant was transferred to a glass vial insert for further analysis.

#### 2.2.4. Chromatographic System

One microliter of the sample was injected via an autosampler (7693A, Agilent Technologies, Palo Alto, CA, USA) into a gas chromatography (GC) system (8890 GC system, Agilent Technologies) coupled with a mass spectrometer (MS, 5977B GC/MSD, Agilent Technologies). The gas chromatographic conditions were as follows: a DB-1MS UI column (Agilent, 30 m × 0.25 mm I.D. and 0.25 μm film thickness) was used. Helium was used as the carrier gas at a constant flow rate of 1 mL/min. A 1 μL volume of sample was injected in a 20:1 split with a split liner (taper, low-pressure drop, with glass wool). The inlet temperature was 250 °C, while the oven temperature was initially held at 150 °C for 1 min and then ramped up to 276 °C at 21 °C/min, then held for 15 min, followed by a post-run time of 3 min at 325 °C. 

The MS was run in a selected ion monitoring (SIM) mode for quantitative analysis, using ion fragments for quantitation and verification as described in Supplementary Table S1. ChemStation software (Agilent, Palo Alto, CA, USA) was used to automatically integrate all peaks and calculate the concentrations of analytes in the injected hexane solution. These data were exported, and the recorded weights of lyophilized feces for each sample were used to calculate analyte concentrations in μg or ng per mg of lyophilized feces. 

To validate the analytical method, linearity was determined by running duplicates of at least 6 concentrations to establish a standard curve for each targeted compound. For the imprecision test, 6 replicates of a pooled sample were assessed in one run for intra-assay variation, and 6 replicates of a random sample were assessed on 6 different days to determine the inter-day variation. The minimum and maximum concentrations of the standard curve, coefficient of correlation (R^2^), curve fit, weighting factor, and the average coefficient of variation (CV%) of the intra- and inter-day precision tests are summarized in Supplementary Table S2. All targeted compounds and their PubChem CID, common name, and IUPAC name are summarized in Supplementary Table S3.

## 3. Results

### 3.1. Study Population

The healthy group consisted of 77 cats (43 male and 34 female) and the CE group of 56 cats (31 male and 25 female). A total of 70 mixed-breed cats and 7 purebred cats (1 each of American Lynx, Bombay, Burmese, Maine Coon, Norwegian Forest, Persian, and Sphynx) were in the healthy group, and 46 mixed-breed cats and 10 purebred cats (3 Siamese; 3 Rag Doll; and 1 each of Abyssinian, Bengal, Birman, and Oriental) were in the CE group. The sex (*p* > 0.999) and frequency of mixed-breed and purebred cats (*p* = 0.19) did not differ between healthy cats and cats with CE. The median age was 8.3 years (range: 0.75–15 years) in healthy cats and was 11 years in cats with CE (range: 2.2–20 years). The median FCEAI score in cats with CE was 6 (range: 2–11) out of a maximum possible score of 19. The most common clinical signs of cats with CE were vomiting (72%) and weight loss (72%), followed by diarrhea (33%), decreased appetite (29%), and lethargy (18%). Among healthy cats, 60 cats (62 cats with a known diet history) were fed a commercial maintenance diet, except for two healthy cats with specialized diets: one on a hydrolyzed protein diet and the other one on a hypoallergenic diet. Among cats with CE, 47 cats with CE (52 cats with a known diet history) were fed a commercial maintenance diet, while 5 cats were on specialized diets: two on a hydrolyzed protein diet and three on a hypoallergenic diet. 

#### Statistical Analysis

All datasets were tested for normal distribution using the Shapiro–Wilk test. Comparisons of sex and breed between cats with CE and healthy cats were evaluated using Fisher’s exact tests. Fecal concentrations of each metabolite in cats with CE and healthy cats were compared using the Mann–Whitney *U* tests. One-way ANOVA was used to compare the fecal concentrations of each targeted metabolite in healthy cats, cats with IBD (CIE), and cats with LGTCL. A post hoc comparison with Dunn’s test was used to identify the differences between groups. Statistical significance was set at *p* < 0.05. Adjusted *p*-values were calculated by the Bonferroni correction method for multiple comparisons, with a multiplication factor of 38 variables for the original *p*-values. A multiple logistic regression model was used to evaluate the potential confounders, including age and sex. The correlations of FCEAI and the concentrations of each targeted metabolite were evaluated by Spearman correlation tests. All statistical analyses were performed in GraphPad Prism 9.0 (GraphPad Software Inc., San Diego, CA, USA). 

### 3.2. Fecal Concentrations of Long-Chain Fatty Acids, Sterols, and Unconjugated Bile Acids

**Figure 1:**
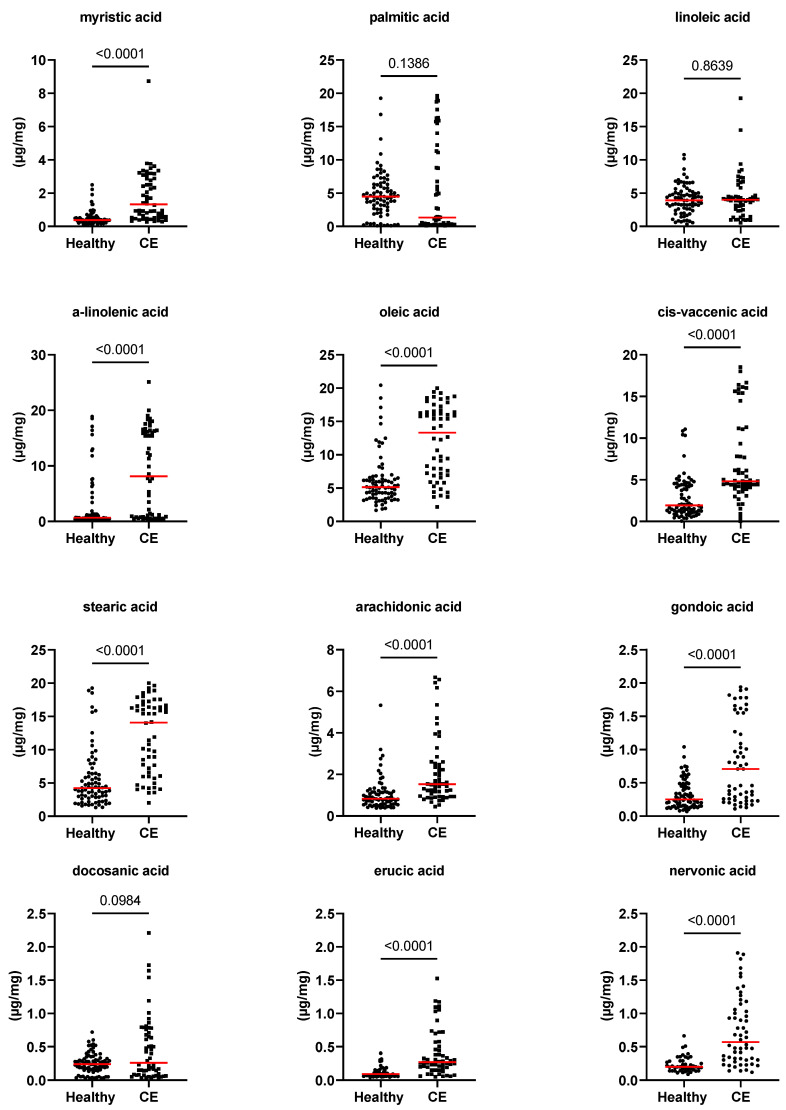
Fecal concentrations of fatty acids (µg/mg) in healthy cats (*n* = 77) and cats with chronic enteropathy (CE, *n* = 56). Red lines represent medians. The *p*-values shown in the figures are unadjusted. For adjusted *p*-values, please refer to Table 1.

**Figure 2:**
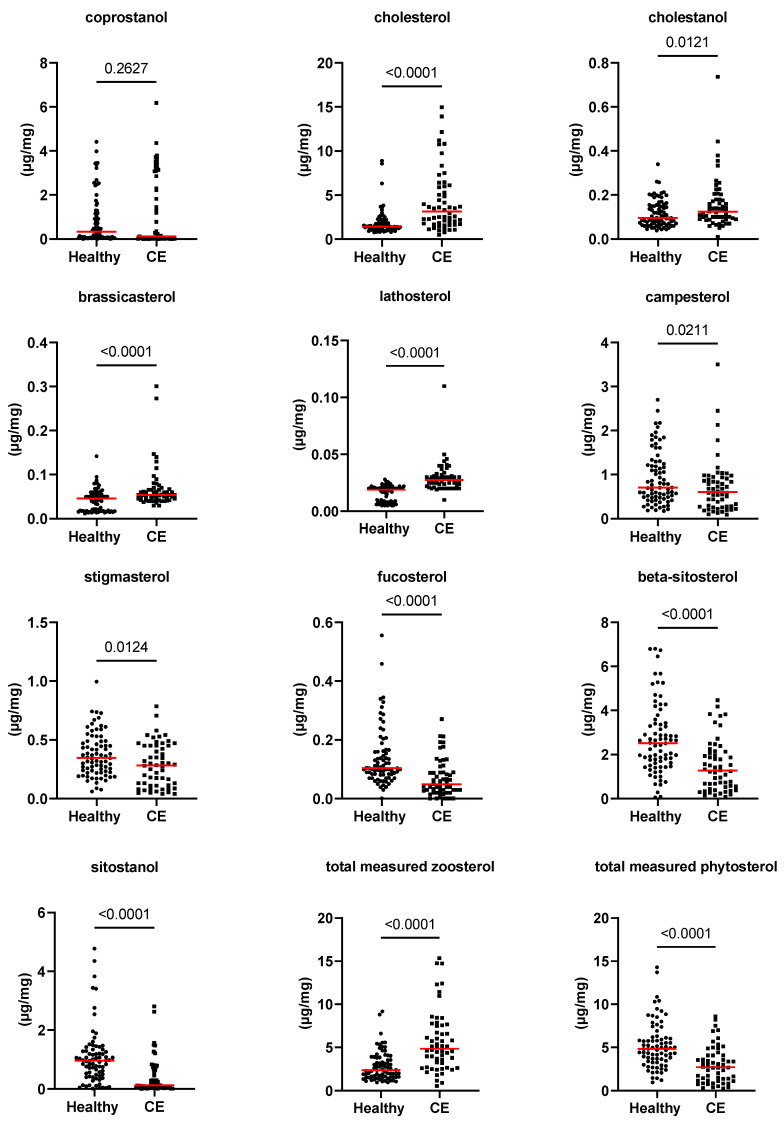
Fecal concentrations of sterols (µg/mg) in healthy cats (*n* = 77) and cats with chronic enteropathy (CE, *n* = 56). Red lines represent medians. The concentration of the total measured zoosterol is the sum of coprostanol, cholesterol, cholestanol, and lathosterol. The concentration of the total measured phytosterol is the sum of the rest measured sterols. The *p*-values shown in the figures are unadjusted. For adjusted *p*-values, please refer to Table 1.

**Figure 3:**
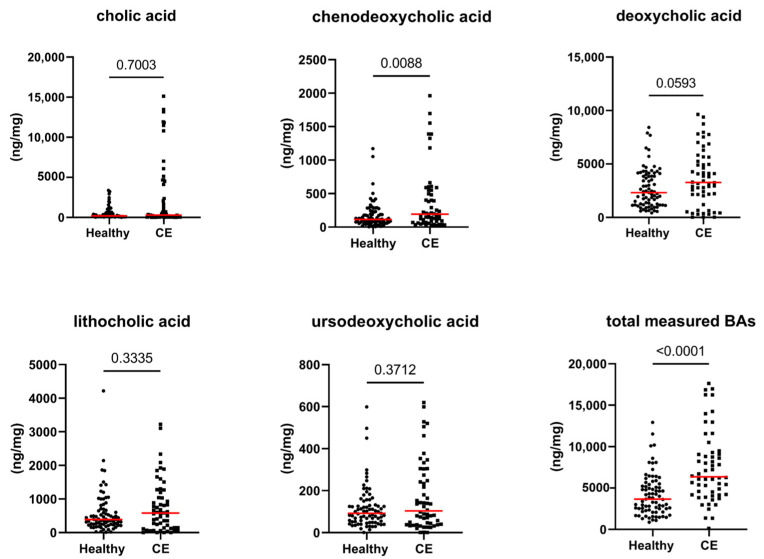
Fecal concentrations of unconjugated bile acids (ng/mg) in healthy cats (*n* = 77) and cats with chronic enteropathy (CE, *n* = 56). Red lines represent medians. The concentration of the total measured bile acids (BAs) is the sum of all measured unconjugated bile acids. The *p*-values shown in the figures are unadjusted. For adjusted *p*-values, please refer to Table 1.

**Figure 4:**
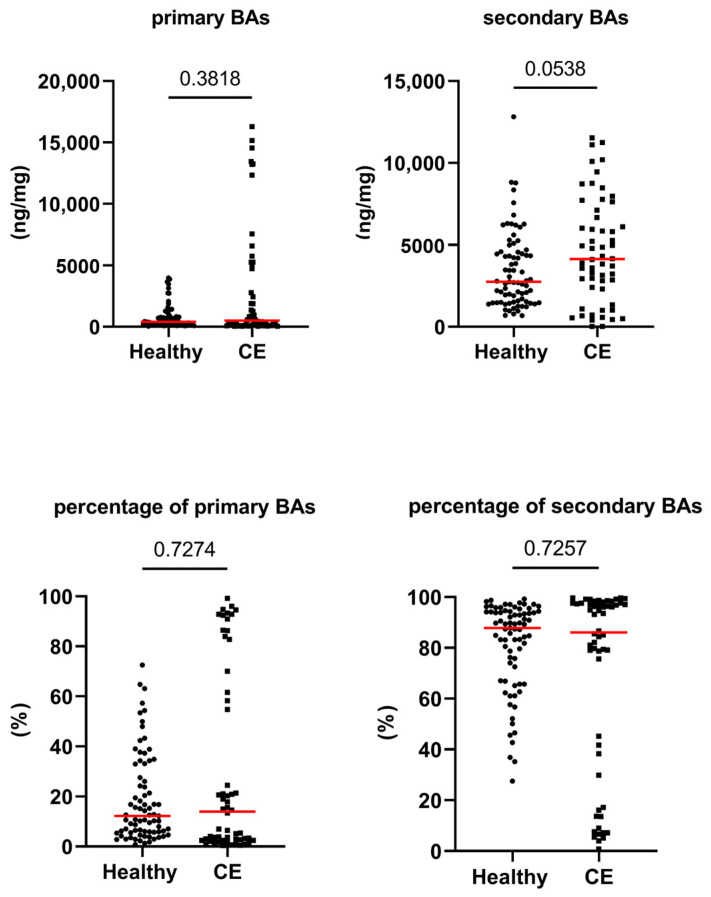
Absolute and relative fecal concentrations of primary and secondary bile acids (BAs) in healthy cats (*n* = 77) and cats with chronic enteropathy (CE, *n* = 56). Red lines represent medians. The concentration of primary BAs is the sum of cholic acid and chenodeoxycholic acid. The concentration of secondary BAs is the sum of lithocholic acid, deoxycholic acid, and ursodeoxycholic acid. The *p*-values shown in the figures are unadjusted. For adjusted *p*-values, please refer to Table 1.

**Figure 5:**
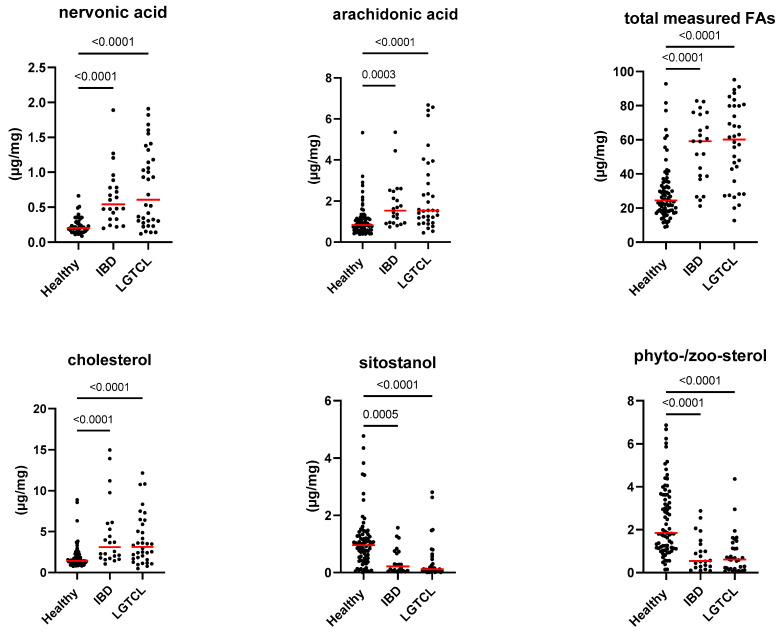
Fecal concentration of selected metabolites: nervonic acid, arachidonic acid, total measured fatty acids (FAs), cholesterol, sitostanol (µg/mg), and the ratio of phytosterol to zoosterol in healthy cats (*n* = 77), cats with inflammatory bowel disease (IBD or CIE, *n* = 22), and cats with low-grade intestinal T-cell lymphoma (LGTCL, *n* = 34). Red lines represent medians. The complete list of targeted metabolites is shown in Supplementary Figure S1. Furthermore, no significant differences between cats with IBD and cats with LGTCL were found in any targeted metabolites.

Figure 1, Figure 2 and Figure 3, as well as Table 1, present the fecal concentrations of the LCFAs, sterols, and BAs in healthy cats and cats with CE. Cats with CE had significantly increased fecal concentrations of nine targeted LCFAs, cholesterol, brassicasterol, lathosterol, and the ratio of cholesterol to coprostanol, compared to the healthy cats. Conversely, significantly decreased fecal concentrations of fucosterol, beta-sitostanol, and sitostanol were observed in cats with CE. Although fecal concentrations of CA did not differ between groups, 25% of cats with CE (*n* = 14) had concentrations above the range of clinically healthy cats (Figure 3). The total measured BAs were significantly increased in cats with CE, with a subset of cats with CE (*n* = 14, 25%) having increased primary BAs (the sum of CA and CDCA divided by the total measured BAs) and decreased secondary BAs (the sum of LCA, DCA, and UDCA divided by the total measured BAs) in feces (Figure 4). After adjusting the *p*-values for multiple comparisons, the differences in the fecal concentrations of cholestanol, campesterol, stigmasterol, and CDCA between the two groups were no longer significant. Fecal concentrations of all targeted compounds did not significantly differ between cats with IBD (CIE) and cats with LGTCL (Figure 5 and Supplementary Figure S1). 

While the multiple logistic regression model exhibited a high accuracy of 97% in distinguishing cats with CE from clinically healthy cats, none of the individual variables (age, sex, or any metabolites) showed statistical significance alone. Additionally, no significant correlations were found between any targeted metabolites and FCEAI. 

### 3.3. Validation of Gas Chromatography with Mass Spectrometry

Supplementary Table S2 shows the validation results of GC-MS method used to quantify targeted lipid compounds. The calibration curves for all targeted analytes showed excellent linearity, with R-squares greater than 99.0. The precision of the method was assessed through intra- and inter-assay variations, expressed as coefficient of variation (CV%). The intra-assay CV% ranged from 0.8% to 6.9%, demonstrating high precision within a single assay run. Similarly, the inter-assay CV% ranged from 1.0% to 12.6%, demonstrating good precision between different assay runs. These results confirmed the reliability and accuracy of the analytical method used in this study.

## 4. Discussion

We developed and validated a quantitative targeted GC-MS method to measure fecal long-chain fatty acids (LCFAs), sterols, and untargeted bile acids (BAs) in cats. Our study revealed significant differences in concentrations of fecal LCFAs, sterols, and BAs between cats with CE and healthy control cats. These findings are consistent with previous untargeted metabolomic studies in cats [7], dogs [24], and humans [9,28,29], suggesting that lipid metabolism is affected in cats with CE.

Concentrations of fecal LCFAs are related to various factors, such as dietary intake, gastrointestinal digestion and absorption of lipids, bile secretion, FA-binding proteins, FA transport protein, and small intestinal transit time, among others [30]. Some specific FAs have been linked to inflammatory activity [31,32,33], making the composition of fecal FAs a potential indicator for gastrointestinal health. In our study, we found increased fecal concentrations of 9 out of 12 targeted LCFAs in cats with CE; these increases might have resulted from maldigestion, malabsorption, inflammation, dysbiosis, and abnormal lipid metabolism.

Excessive loss might significantly contribute to the increased fecal concentrations of FAs observed in our study. One explanation might be the damaged intestinal mucosa, which can lead to decreased FA receptors. The increased fecal concentration of nervonic acid in cats with CE may indicate deeper mucosal damage in the intestine, as nervonic acid is an essential component of myelin sheath in nerve tissues. The nervous system is mainly located in the muscle layer of the intestine. Therefore, the increased fecal concentration of nervonic acid could be due to more extensive or deep intestinal damage in cats with CE. Nervonic acid can be synthesized from oleic acid or obtained through direct digestion. As most cats were on a maintenance diet and these diets were similar between healthy cats and cats with CE, it was unlikely that the increased fecal concentration of nervonic acid can be solely attributed to dietary factors. A recent study using a murine colitis model suggested that oral supplementation of nervonic acid could potentially restore intestinal barrier function by suppressing the NF-κB signaling pathway [34]. This provides an additional perspective on how the loss of nervonic acid in cats with CE might contribute to the alteration in intestinal barrier function. Moreover, the increased fecal concentrations of saturated LCFAs have been linked to increase colonic motility in a murine model of long-term colonic dysfunction [35]. This increased motility rate may contribute to the presence of diarrhea in some cats with CE. The increased motility rate might decrease the time for digestion and absorption, leading to a vicious cycle. Among the 18 cats with CE with diarrhea, twelve had a concentration of total measured FA above 67 µg/mg (the 95th percentile observed in the healthy control cats). Additionally, the loss of FAs might lead to decreased serum concentrations of FAs, which is evident in humans with IBD with decreased serum concentrations of nervonic acid, arachidonic acid, and palmitic acid [36]. Similarly, one study also reported on decreased concentrations of oleic acid, stearic acid, and saturated FAs in serum from dogs with CE using a canine-specific proton nuclear magnetic resonance (^1^H NMR) spectroscopy platform [37].

Gut inflammation is also commonly found in cats with CE, which might be an explanation of the increased fecal concentrations of arachidonic acid in cats with CE. As arachidonic acid is the precursor of prostaglandin and leukotriene, which are associated with an inflammatory response [38]. Additionally, arachidonic acid is unlikely to be affected by the diet, suggesting an active inflammatory response in the gastrointestinal tract in cats with CE.

Diet intervention is a strategy to manage cats [39,40] and dogs [41,42] with CE, as well as in humans. In a prospective study of 412 human patients with ulcerative colitis in remission, high dietary intakes of myristic acid and alpha-linolenic acid were associated with an increased risk of flares in these patients [43]. Additionally, fecal concentrations of lauric acid and myristic acid were decreased in patients with ulcerative colitis after low-fat dietary intervention [44], which suggests that a dietary intervention might be crucial to maintaining remission. Future investigations into the modulation of FAs in diet to manage cats with CE are warranted.

The sterols targeted in this study can be classified into two major groups: zoosterols (sterols of animal origin) and phytosterols (sterols of plant origin). Interestingly, in cats with CE, fecal concentrations of zoosterols were higher, and concentrations of most phytosterols were lower than those in healthy controls. These findings are consistent with the results of our previous untargeted study [7]. The mechanisms driving these changes are not yet well understood, but the reduced phytosterols and increased zoosterols concentrations in cats with CE may result from several factors, including inflammation-associated metabolic changes, shifts in bacterial metabolism, and potential disturbances in phytosterol-producing and cholesterol-transforming bacterial populations. In humans with active Crohn’s disease, serum concentrations of total cholesterol, low-density lipoprotein cholesterol, and high-density lipoprotein cholesterol were lower, compared to the healthy controls [45], indicating an excessive loss of zoosterols in feces. Although decreased fecal concentrations of phytosterols were not widely reported in humans with IBD, phytosterols and their derivatives have been shown to modulate inflammation, possess antioxidant and antimicrobial effects, and have immunomodulatory function in in vitro studies and in animal models [46]. Because of the similar structure of phytosterols and cholesterol, phytosterols can inhibit the absorption of cholesterol and further reduce serum cholesterol levels. In a dextran sodium sulfate (DSS)-induced murine colitis model, mice with oral stigmasterol administration had a delayed onset of colitis, a reduced pathohistological score in the colon; and a downregulated expression of inflammatory factors, including IL-6, IL-1β, and TNF-α [47]. In another study, the oral administration of β-sitosterol decreased concentrations of proinflammatory cytokines and plasma endotoxins in mice with high-fat diets [48]. Investigations into adding phytosterols to the diets of cats with CE are warranted.

While previous research has found increased fecal concentrations of cholesterol and coprostanol (the end product of bacterial metabolism of cholesterol) in humans with colon cancer and adenomatous polyps [49], we did not find increased fecal concentrations of coprostanol in cats with CE. However, the cholesterol-to-coprostanol ratio was significantly higher in cats with CE than in control cats. This finding was similar to previous studies in patients with Crohn’s disease, ulcerative colitis, or *Clostridium difficile* infection that had lower rates of conversion of cholesterol to coprostanol [29,50]. Our finding of a significantly increased cholesterol-to-coprostanol ratio in the fecal samples from cats with CE indicates a decreased abundance of cholesterol-metabolizing bacteria, for example, *Bacteroides*, in cats with CE. *Bacteroides dorei* D8 have been found to reduce fecal cholesterol by transforming it into coprostanol in humans [51]. Similar to studies in humans with IBD [52], the decreased fecal abundance of *Bacteroides* in cats with CE [4] suggests that *Bacteroides* might be involved in the metabolism of cholesterol in cats. 

Cats with CE did not have increased fecal CA or decreased absolute concentration of secondary BAs. Notably, 25% of cats with CE had increased total fecal BAs and increased fecal primary BAs and decreased secondary BAs. This finding probably resulted from malabsorption of BAs and intestinal dysbiosis. This malabsorption of BAs might have resulted from reduced ileal protein expression of apical sodium-dependent bile acid transporter (ASBT), as observed in humans with Crohn’s disease [53]. However, to our knowledge, there are no studies on the distribution of ASBT in the feline gastrointestinal tract and whether ASBT is downregulated in cats with CE. Inflammation and shortened ileal villi might also contribute to the malabsorption of BAs [54,55]. When an excessive amount of primary BAs enter the colon, it can increase intestinal motility, leading to a reduction in transit time [56]. This rapid transit may limit the opportunity for bacteria to convert primary BAs into secondary BAs. Among 16 cats with increased total fecal BAs, 15 cats had a fecal abundance of *P. hiranonis* below the reference interval [4]. Additionally, the significant correlation between the fecal abundance of *P. hiranonis* and percentage of secondary BAs further supports the idea that *P. hiranonis* might be the major BA converter in cats, as has been described for dogs [24] and humans with inflammatory bowel disease [57]. The decreased abundance of *P. hiranonis* in cats with CE [4] might be one of the reasons for the increased levels of primary BAs. Collectively, a decreased excretion of secondary BAs may be attributed to a reduced transit time, reduced fecal pH [58], and impaired 7-alpha-dehydroxylase activity (i.e., the decreased abundance of *P. hiranonis*) [57].

Although this study identified significant differences in fecal concentrations of several LCFAs and sterols between healthy control cats and cats with CE, there was considerable overlap between groups and high variation among individuals. Notably, the interindividual variation appeared visually wider in cats with CE, compared to the healthy control cats. While the severity of clinical signs and the specific type of CE could contribute to this finding, no significant correlations were found between any targeted compounds and FCEAI. Moreover, the concentrations of targeted metabolites did not significantly differ between cats with IBD (CIE) and cats with LGTCL, which was consistent with the findings of an untargeted metabolomic study [7]. These findings indicate that no single FA or sterol can be used to differentiate cats with CE from healthy cats or to distinguish between cats with IBD (CIE) and those with LGTCL. However, the multiple logistic regression model, including all targeted metabolites, showed a high accuracy of 97% in predicting cats with CE from healthy cats. Investigation in a combination of several metabolites to assess the severity of the disease might be warranted. For example, fecal fatty acid profiling has been developed to identify human patients with colorectal cancer [23]. 

Several limitations of this study should be noted. Firstly, due to the nature of a retrospective study design, the diet was not standardized and not available in 15 healthy cats and 4 cats with CE, and the effect of diet on the targeted metabolites could not be excluded. The reason why the owner of healthy cats chose a specialized diet was unknown. However, for the cats with CE, a specialized diet might be one part of their treatment. However, in this study, most of healthy cats and cats with CE were consuming commercial feline maintenance diets. Only two healthy cats and five cats with CE were on a specialized diet. Moreover, a similar pattern of changes in these targeted fecal metabolites was also observed in dogs with chronic enteropathy [59] and untargeted metabolome in cats with CE [7], where the included subjects were also with various diets. Therefore, it is unlikely that the diet had a significant impact on the metabolites compared to the effect of the disease. Secondly, fecal samples were collected only once from each cat, and it was not possible to assess whether the concentrations of targeted compounds changed in response to therapy. Longitudinal fecal sampling from cats with CE is warranted to evaluate the prognostic utility of the targeted compounds. Thirdly, the analytical method used in this study did not allow the quantification of conjugated BAs. Increased concentrations of serum [60] and fecal conjugated primary BAs and 3-OH-sulfated BAs have been observed in humans with IBD [57,60]. Additionally, conjugated UDCA has been shown to prevent colitis-related dysbiosis in a murine model [61]. Measuring both conjugated and unconjugated BAs in the future could help understand the role of BAs in the pathogenesis of CE in cats. Fourthly, fecal samples collected within 24 h after defecation might have an impact on metabolic profiles. However, a study found that samples stored at room temperature for 52 h had a minimal change compared to individual variability [62]. Lastly, the study did not consider the impact of age, geography, or previous antibiotic use. These factors could contribute to the observed variability and highlight areas for future research. 

## 5. Conclusions

In conclusion, our findings suggest that cats with CE have a dysmetabolism of lipids, including LCFAs, sterols, and unconjugated BAs. A comprehensive profile incorporating the levels of multiple LCFAs, sterols, and BAs may potentially be useful to understand intestinal dysfunction in feline CE. Investigation into the relationship between microbial composition and metabolic shift is warranted. 

## Figures and Tables

**Table 1:** Fecal concentrations of targeted compounds in healthy cats and cats with chronic enteropathy (CE). Comparisons between groups were analyzed by Mann–Whitney *U* tests. Bolded *p*- or q-values indicate statistical significance. An adjusted *p*-value is calculated by the Bonferroni correction method. BA = bile acid, FA = fatty acid, (Z) = zoosterol, and (P) = phytosterol.

Variable	Healthy Control (*n* = 77) Median [Range]	Cats with CE (*n* = 56) Median [Range]	*p*-Value	Adjusted *p*-Value
myristic acid (µg/mg)	0.4 [0.1–2.5]	1.3 [0.3–8.7]	**<0.0001**	**0.004**
palmitic acid (µg/mg)	4.5 [0.1–19.3]	1.3 [0.1–19.6]	0.139	1.000
linoleic acid (µg/mg)	3.9 [0.4–10.8]	4 [0.5–19.3]	0.864	1.000
alpha-linolenic acid (µg/mg)	0.7 [0.2–18.9]	8.2 [0.2–25.1]	**<0.0001**	**0.004**
oleic acid (µg/mg)	5.1 [1.7–20.5]	13.3 [2.2–20]	**<0.0001**	**0.004**
cis-vaccenic acid (µg/mg)	2 [0–43.3]	4.8 [0–18.5]	**<0.0001**	**0.004**
stearic acid (µg/mg)	4.2 [1.3–19.3]	14.1 [2–20]	**<0.0001**	**0.004**
arachidonic acid (µg/mg)	0.8 [0.4–5.3]	1.5 [0.5–6.7]	**<0.0001**	**0.004**
gondoic acid (µg/mg)	0.3 [0.1–1]	0.7 [0.1–1.9]	**<0.0001**	**0.004**
erucic acid (µg/mg)	0.1 [0–0.4]	0.3 [0.1–1.5]	**<0.0001**	**0.004**
behenic acid (µg/mg)	0.2 [0–0.7]	0.3 [0–2.2]	0.098	1.000
nervonic acid (µg/mg)	0.2 [0–0.7]	0.6 [0.1–1.9]	**<0.0001**	**0.004**
coprostanol (Z) (µg/mg)	0.3 [0–4.4]	0.1 [0–6.2]	0.263	1.000
cholesterol (Z) (µg/mg)	1.4 [0.8–8.9]	3.1 [0.5–15]	**<0.0001**	**0.004**
cholestanol (Z) (µg/mg)	0.1 [0–0.3]	0.1 [0–0.7]	**0.012**	0.456
brassicasterol (P) (µg/mg)	0.05 [0.01–0.14]	0.05 [0.03–0.3]	**<0.0001**	**0.004**
lathosterol (Z) (µg/mg)	0.02 [0–0.03]	0.03 [0.01–0.11]	**<0.0001**	**0.004**
campesterol (P) (µg/mg)	0.7 [0.2–2.7]	0.6 [0.1–3.5]	**0.021**	1.000
stigmasterol (P) (µg/mg)	0.3 [0.1–1]	0.3 [0–0.8]	**0.012**	1.000
beta-sitosterol (P) (µg/mg)	2.5 [0–6.8]	1.3 [0.1–4.5]	**<0.0001**	**0.004**
fucosterol (P) (µg/mg)	0.1 [0–0.6]	0 [0–0.3]	**<0.0001**	**0.004**
sitostanol (P) (µg/mg)	1 [0–4.8]	0.1 [0–2.8]	**<0.0001**	**0.004**
cholic acid (ng/mg)	197 [6–3368]	290 [9–113]	0.700	1.000
chenodeoxycholic acid (ng/mg)	113 [6–1172]	192 [20–1961]	**0.009**	0.342
lithocholic acid (ng/mg)	389 [11–4218]	580 [1–3222]	0.334	1.000
deoxycholic acid (ng/mg)	2315 [419–8420]	3266 [15–9635]	0.059	1.000
ursodeoxycholic acid (ng/mg)	92 [1–599]	103 [1–619]	0.371	1.000
total primary BAs (ng/mg)	394 [35–3974]	503 [39–16,295]	0.382	1.000
total secondary BAs (ng/mg)	2740 [682–12,822]	4136 [17–11,534]	0.054	1.000
total measured BAs (ng/mg)	3664 [867–12,920]	6353 [125–17,620]	**<0.0001**	**0.004**
primary BAs (%)	12 [1–73]	14 [0–99]	0.727	1.000
secondary BAs (%)	88 [27–99]	86 [1–100]	0.726	1.000
total measured FAs (µg/mg)	24 [9–93]	59 [13–95]	**<0.0001**	**0.004**
total measured sterols (µg/mg)	7 [4–19]	8 [1–23]	0.280	1.000
phytosterol (µg/mg)	4.8 [1–14.3]	2.7 [0.3–8.6]	**<0.0001**	**0.004**
zoosterol (µg/mg)	2.4 [1–9.2]	4.8 [0.5–15.4]	**<0.0001**	**0.004**
phytosterol/zoosterol	1.8 [0.1–6.9]	0.6 [0–4.4]	**<0.0001**	**0.004**
cholesterol/coprostanol	4.6 [0.2–959.2]	26.5 [0.2–8452]	**0.0156**	0.593

## Data Availability

Not applicable.

## Supplementary Materials

The following supporting information can be downloaded at https://www.mdpi.com/article/10.3390/ani13172753/s1: Figure S1. Targeted compounds in healthy cats, cats with IBD (CIE), and cats with LGTCL. Table S1. Targeted compounds in the GC-MS assay and the characteristics of the ion fragments used for quantification and qualification. Table S2. The validation results of the GC-MS method to quantify targeted lipid compounds. Table S3. PubChem CID, common name, and IUPAC name of targeted compounds.
