# Supplementary material for: Fecal Concentrations of Long-Chain Fatty Acids, Sterols, and Unconjugated Bile Acids in Cats with Chronic Enteropathy
Source: Animals (Basel). 2023 Aug 30;13(17):2753. doi: 10.3390/ani13172753 (PMC10486672; doi:10.3390/ani13172753)
Supplement: Supplementary file 1 [file animals-13-02753-s001.zip › animals-2550834-supplementary.pdf]

Compound	Retention time (min)	Quantitation ions (nominal m/z)	Qualifying ions (nominal m/z [% intensity])
myristic acid (14:0)	5.9	229	56[225], 185[28], 271[55]
palmitic acid (16:0)	6.8	257	312[20], 239[53]
linoleic acid (18:2, n-6)	7.6	263	262[58], 336[26]
oleic acid (18:1, n-9)	7.6	264	265[98], 222[41], 79[73]
cis-vaccenic acid (18:1, n-7)	7.6	265	264[86], 338[12], 79[116]
alpha-linolenic acid (18:3, n-3)	7.7	261	79[381], 334[67]
d_4-stearic acid (ISTD)	7.7	289	344[35], 271[48]
stearic acid (18:0)	7.7	285	340[33]
arachidonic acid (20:4, n-6)	8.4	292	150[1931],203[20]
gondoic acid (20:1, n-9)	8.7	293	292[144], 250[34], 366[12]
erucic acid (22:1, n-9)	10.1	321	320[105], 236[28], 394[93]
behenic acid (22:0)	10.3	341	396[63], 323[43]
d_4-cholestane (ISTD)	11.4	361	221[218], 376[76]
nervonic acid (24:1, n-9)	12.3	348	349[88]
coprostanol	14.1	370	355[33], 257[21]
d_6-cholesterol (ISTD)	15.7	464	334[253], 359[70], 374[220]
cholesterol	15.8	368	353[47], 329[117]
cholestanol	16.0	445	460[74], 355[87]
brassicasterol	16.7	TIC	470[23], 380[38], 341[23]
lathosterol	17.0	458	443[26], 213[47]
campesterol	18.1	343	382[87], 367[42]
stigmasterol	18.9	484	394[140], 469[21]
β-sitosterol	20.4	357	396[99], 381[43]
fucosterol	20.5	386	296[78]
d_7-sitostanol (ISTD)	20.6	480	390[59], 405[35]
sitostanol	20.8	473	488[77], 383[85]
d_4-lithocholic acid (ISTD)	23.4	219	261[77]
lithocholic acid	23.4	215	257[92]
deoxycholic acid	25.2	255	256[21], 250[16]
d_4-cholic acid (ISTD)	26.1	257	414[90]
chenodeoxycholic acid	26.0	412	255[80], 397[28]
cholic acid	26.1	253	410[62], 500[31]
ursodeoxycholic acid	27.5	502	503[31]

Supplementary Table S1. Targeted compounds in the GC-MS assay and the characteristics of the ion fragments used for quantification and qualification, ordered by increasing retention time. Number of carbons, number of double bonds, and position of double bonds were specified for fatty acids. ISTD: deuterated internal standard.

Compound	Min (μg/ mL)	Max (μg/ mL)	R^2	curve fit and weighting factor	intra-assay variation CV%	inter-assay variation CV%
myristic acid	1.6	200	99.9	QE	1.7	2.4
palmitic acid	3.9	1000	99.4	QE	1.1	1.2
linoleic acid	7.8	1000	99.7	QI	1.9	1.0
oleic acid	7.8	1000	99.0	QI	4.8	4.1
cis-vaccenic acid	3.1	400	99.4	QI	1.6	4.9
alpha-linolenic acid	0.8	200	99.9	QE	4.0	10.0
stearic acid	7.8	1000	99.8	QI	1.1	1.1
arachidonic acid	3.1	400	99.9	QE	3.0	4.9
gondoic acid	1.6	100	99.9	QI	2.2	3.8
erucic acid	0.8	100	99.9	QI	2.8	7.5
behenic acid	1.6	200	99.9	QI	6.9	7.6
nervonic acid	1.6	200	99.9	QI	4.3	10.1
coprostanol	0.8	200	99.9	QI	3.5	5.2
cholesterol	3.1	800	99.9	QI	1.3	1.4
cholestanol	0.8	100	99.9	QE	2.4	3.2
brassicasterol	0.8	100	99.9	QI	2.6	3.9
lathosterol	0.8	50	99.7	QI	2.6	1.2
campesterol	1.6	200	99.9	QI	1.7	4.6
stigmasterol	1.6	100	99.9	QI	2.4	6.1
β-sitosterol	6.3	400	99.9	QE	1.7	1.1
fucosterol	0.8	100	99.9	QE	1.3	1.0
sitostanol	1.6	200	99.9	LI	1.6	2.2
lithocholic acid	2.0	250	99.8	QI	4.9	12.4
deoxycholic acid	4.0	500	99.9	QE	3.8	12.6
chenodeoxycholic acid	0.8	100	99.9	QI	4.3	9.3
cholic acid	2.0	800	99.9	QI	0.8	6.1
ursodeoxycholic acid	0.4	50	99.6	QE	5.6	11.9

Supplementary Table S2. The validation results of GC-MS method to quantify targeted lipid compounds. min: minimum concentrations of the standard curve; max: maximum concentrations of the standard curve; Q: quadratic regression; L: linear regression; E: equal weighting; I: inverse of concentration weighting.

Compound	PubChem CID	Common name	IUPAC name
myristic acid (14:0)	11005	n-tetradecanoic acid, crodacid	tetradecanoic acid
palmitic acid (16:0)	985	cetylic acid	hexadecanoic acid
linoleic acid (18:2, n-6)	5280450	linolic acid, telfairic acid	(9Z,12Z)-octadeca-9,12-dienoic acid
oleic acid (18:1, n-9)	445639	cis-9-octadecenoic acid, oleate	(Z)-octadec-9-enoic acid
cis-vaccenic acid (18:1, n-7)	5282761	cis-11-octadecenoic acid, asclepic acid	(Z)-octadec-11-enoic acid
alpha-linolenic acid (18:3, n-3)	5280934	linolenic acid, linolenate	(9Z,12Z,15Z)-octadeca-9,12,15-trienoic acid
stearic acid (18:0)	5281	n-octadecanoic acid, stearophanic acid	octadecanoic acid
arachidonic acid (20:4, n-6)	444899	arachidonate, immunocytophyte	(5Z,8Z,11Z,14Z)-icosa-5,8,11,14-tetraenoic acid
gondoic acid (20:1, n-9)	5282768	cis-11-eicosenoic acid, 11-eicosenoic acid	(Z)-icos-11-enoic acid
erucic acid (22:1, n-9)	5281116	cis-13-docosenoic acid, 13-docosenoic acid	(Z)-docos-13-enoic acid
behenic acid (22:0)	8215	1-docosanoic acid	docosanoic acid
nervonic acid (24:1, n-9)	5281120	cis-15-tetracosenoic acid, selacholeic acid	(Z)-tetracos-15-enoic acid
coprostanol	221122	coprosterol, stercorin	(3S,5R,8R,9S,10S,13R,14S,17R)-10,13-dimethyl-17-[(2R)-6-methylheptan-2-yl]-2,3,4,5,6,7,8,9,11,12,14,15,16,17-tetradecahydro-1H-cyclopenta[a]phenanthren-3-ol
cholesterol	5997	cholesterin, cholestrin	(3S,8S,9S,10R,13R,14S,17R)-10,13-dimethyl-17-[(2R)-6-methylheptan-2-yl]-2,3,4,7,8,9,11,12,14,15,16,17-dodecahydro-1H-cyclopenta[a]phenanthren-3-ol
cholestanol	6665	dehydrocholesterol, beta-cholestanol, zymostanol	(3S,5S,8R,9S,10S,13R,14S,17R)-10,13-dimethyl-17-[(2R)-6-methylheptan-2-yl]-2,3,4,5,6,7,8,9,11,12,14,15,16,17-tetradecahydro-1H-cyclopenta[a]phenanthren-3-ol
brassicasterol	5281327	brassicasterin	(3S,8S,9S,10R,13R,14S,17R)-17-[(E,2R,5R)-5,6-dimethylhept-3-en-2-yl]-10,13-dimethyl-2,3,4,7,8,9,11,12,14,15,16,17-dodecahydro-1H-cyclopenta[a]phenanthren-3-ol
lathosterol	65728	gamma-cholesterol	(3S,5S,9R,10S,13R,14R,17R)-10,13-dimethyl-17-[(2R)-6-methylheptan-2-yl]-2,3,4,5,6,9,11,12,14,15,16,17-dodecahydro-1H-cyclopenta[a]phenanthren-3-ol
campesterol	173183	campesterin	(3S,8S,9S,10R,13R,14S,17R)-17-[(2R,5R)-5,6-dimethylheptan-2-yl]-10,13-dimethyl-2,3,4,7,8,9,11,12,14,15,16,17-dodecahydro-1H-cyclopenta[a]phenanthren-3-ol
stigmasterol	5280794	beta-stigmasterol, stigmasterin	(3S,8S,9S,10R,13R,14S,17R)-17-[(E,2R,5S)-5-ethyl-6-methylhept-3-en-2-yl]-10,13-dimethyl-2,3,4,7,8,9,11,12,14,15,16,17-dodecahydro-1H-cyclopenta[a]phenanthren-3-ol
β-sitosterol	222284	sitosterol, cupreol, azuprostat	(3S,8S,9S,10R,13R,14S,17R)-17-[(2R,5R)-5-ethyl-6-methylheptan-2-yl]-10,13-dimethyl-2,3,4,7,8,9,11,12,14,15,16,17-dodecahydro-1H-cyclopenta[a]phenanthren-3-ol
fucosterol	5281328	fucosterin, (24e)-24-n-propylidenecholesterol	(3S,8S,9S,10R,13R,14S,17R)-10,13-dimethyl-17-[(E,2R)-5-propan-2-ylhept-5-en-2-yl]-2,3,4,7,8,9,11,12,14,15,16,17-dodecahydro-1H-cyclopenta[a]phenanthren-3-ol
sitostanol	241572	stigmastanol, fucostanol, spinastanol	(3S,5S,8R,9S,10S,13R,14S,17R)-17-[(2R,5R)-5-ethyl-6-methylheptan-2-yl]-10,13-dimethyl-2,3,4,5,6,7,8,9,11,12,14,15,16,17-tetradecahydro-1H-cyclopenta[a]phenanthren-3-ol
lithocholic acid	9903	3alpha-hydroxy-5beta-cholan-24-oic acid	(4R)-4-[(3R,5R,8R,9S,10S,13R,14S,17R)-3-hydroxy-10,13-dimethyl-2,3,4,5,6,7,8,9,11,12,14,15,16,17-tetradecahydro-1H-cyclopenta[a]phenanthren-17-yl]pentanoic acid
deoxycholic acid	222528	deoxycholate, desoxycholic acid, cholerebic	(4R)-4-[(3R,5R,8R,9S,10S,12S,13R,14S,17R)-3,12-dihydroxy-10,13-dimethyl-2,3,4,5,6,7,8,9,11,12,14,15,16,17-tetradecahydro-1H-cyclopenta[a]phenanthren-17-yl]pentanoic acid
chenodeoxycholic acid	10133	chenodiol, chenic acid	(4R)-4-[(3R,5S,7R,8R,9S,10S,13R,14S,17R)-3,7-dihydroxy-10,13-dimethyl-2,3,4,5,6,7,8,9,11,12,14,15,16,17-tetradecahydro-1H-cyclopenta[a]phenanthren-17-yl]pentanoic acid
cholic acid	221493	cholate, cholalic acid	(4R)-4-[(3R,5S,7R,8R,9S,10S,12S,13R,14S,17R)-3,7,12-trihydroxy-10,13-dimethyl-2,3,4,5,6,7,8,9,11,12,14,15,16,17-tetradecahydro-1H-cyclopenta[a]phenanthren-17-yl]pentanoic acid
ursodeoxycholic acid	31401	ursodiol, actigall, ursodeoxycholate	(4R)-4-[(3R,5S,7S,8R,9S,10S,13R,14S,17R)-3,7-dihydroxy-10,13-dimethyl-2,3,4,5,6,7,8,9,11,12,14,15,16,17-tetradecahydro-1H-cyclopenta[a]phenanthren-17-yl]pentanoic acid

Supplementary Table S3. Targeted compounds in the GC-MS assay and their PubChem CID, common name, and IUPAC name.

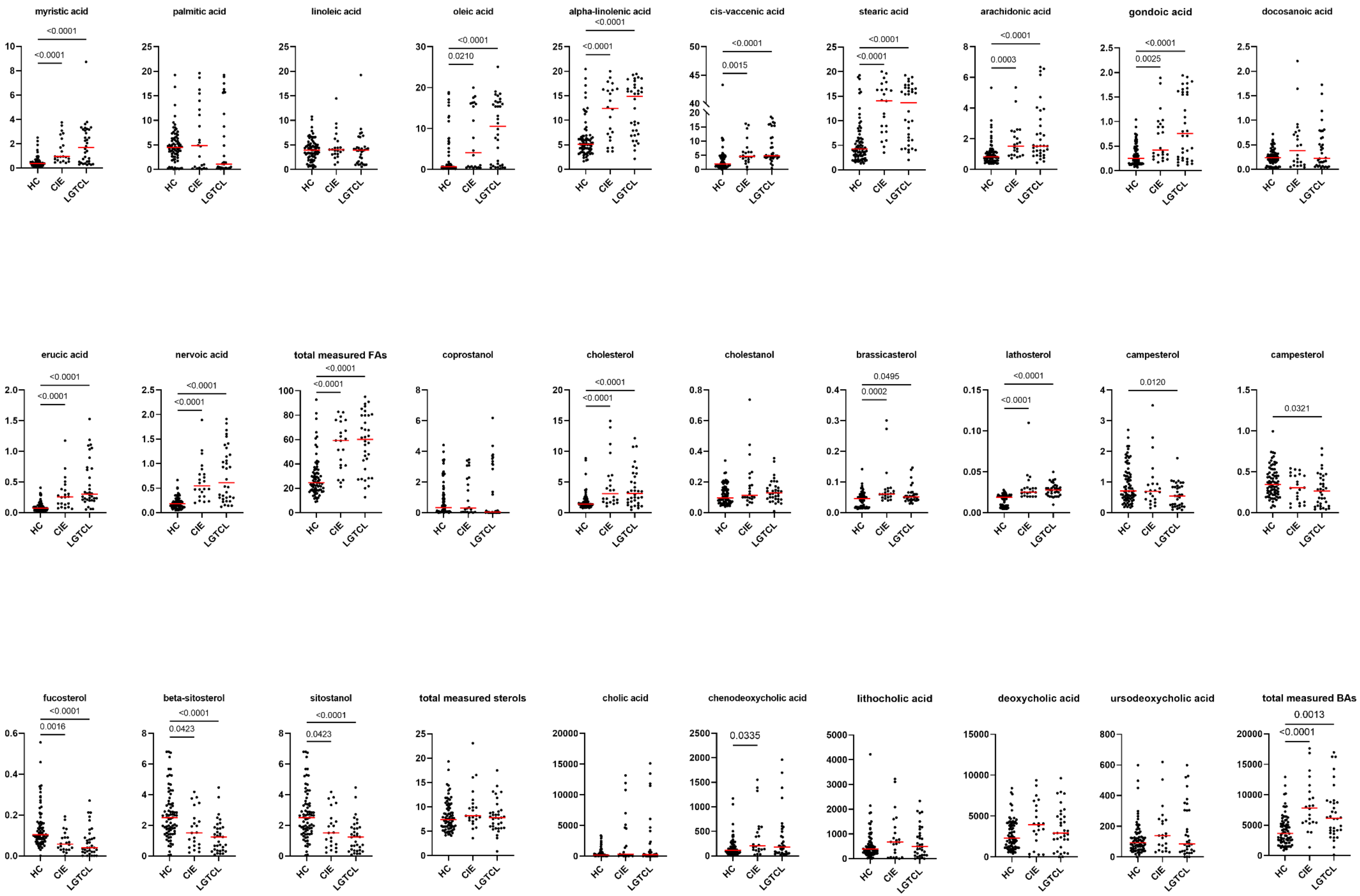

Supplementary Figure S1. Fecal concentrations of targeted compounds in healthy cats (HC), cats with chronic inflammatory enteropathy (CIE, IBD), and cats with low-grade intestinal T-cell lymphoma (LGTCL). Red lines represent medians. Unit for fatty acids and sterols (μg/mg); unit for bile acids (ng/mg). The p-values shown in the figures are unadjusted P-values.
